# Utility of the Iodine Overlay Technique and Virtual Nonenhanced Images for the Preoperative T Staging of Colorectal Cancer by Dual-Energy CT with Tin Filter Technology

**DOI:** 10.1371/journal.pone.0113589

**Published:** 2014-12-03

**Authors:** Chiao-Yun Chen, Jui-Sheng Hsu, Twei-Shiun Jaw, Deng-Chyang Wu, Ming-Chen Paul Shih, Chien-Hung Lee, Chao-Hung Kuo, Yi-Ting Chen, Ming-Lai Lai, Gin-Chung Liu

**Affiliations:** 1 Graduate Institute of Medicine, College of Medicine, Kaohsiung Medical University, Kaohsiung, Taiwan; 2 Department of Radiology, Faculty of Medicine, College of Medicine, Kaohsiung Medical University, Kaohsiung, Taiwan; 3 Department of Medical Imaging, Kaohsiung Medical University Hospital, Kaohsiung, Taiwan; 4 Kaohsiung Municipal Hsiao-Kang Hospital, Kaohsiung, Taiwan; 5 Department of Medicine, Faculty of Medicine, College of Medicine, Kaohsiung Medical University, Kaohsiung, Taiwan; 6 Department of Public Health, Kaohsiung Medical University, Kaohsiung, Taiwan; 7 Division of Gastroenterology, Department of Internal Medicine, Kaohsiung Medical University Hospital, Kaohsiung, Taiwan; 8 Department of Pathology, Kaohsiung Medical University Hospital, Kaohsiung, Taiwan; University of Nebraska Medical Center, United States of America

## Abstract

**Objectives:**

To evaluate the diagnostic accuracy and the potential radiation dose reduction of dual-energy CT (DECT) for tumor (T) staging of colorectal cancer (CRC) using iodine overlay (IO) and virtual nonenhanced (VNE) images.

**Materials and Methods:**

This retrospective study included 103 consecutive patients who underwent nonenhanced CT and enhanced DECT for preoperative CRC staging. Enhanced weighted-average (WA), IO and VNE images were reconstructed from enhanced 80 kVp and Sn140 kVp scans. Two radiologists assessed image qualities of the true nonenhanced (TNE) and VNE images. For T-staging, another two radiologists independently interpreted all scans in two separate reading sessions: in the first session, only images derived from the single phase DECT acquisition (IO and VNE images) were read. In the second reading session after 30 to 50 (average:42) days, the same assessment was again performed with the TNE and enhanced WA images thereby simulating conventional dual-phase single-energy CT. The tumor node metastasis (TNM) system was used for staging with histopathologic reports as gold standard. Analysis of variance was used for statistical analysis.

**Results:**

The signal-to-noise ratios (SNRs) of the tumors and normal reference tissues showed significant correlation between the TNE and VNE images (*P*<0.01). The mean iodine overlay value (48.4 HU±12.2) and enhancement (49.4 HU±11.8) value of CRCs had no significant difference (P = 0.52).The mean image noise on TNE (5.0±1.1) and VNE (5.3±1.1) images were similar (*P* = 0.07). The quantitative qualities of the VNE images were mildly inferior to the TNE images. Overall accuracy of T-stage CRC when using single-phase acquisition was slightly better than the dual-phase acquisition (90.3% vs 87.4%) (*P* = 0.51). The mean dose of the single-phase DECT acquisition was 6.2mSv comparing with 14.3mSv of dual-phase.

**Conclusion:**

Single-phase DECT using IO and VNE images yields a high accuracy in T-staging of CRCs. Thereby, the radiation exposure of the patients can be reduced.

## Introduction

The prognosis of patients with colorectal cancer (CRC) depends on the stage of disease at the time of diagnosis [1]. Computed tomography (CT) is a robust method for stratifying patients preoperatively, with similar accuracy as histopathology for predicting outcome of the patients [2]. Accurate preoperative staging is essential for the planning of optimal therapy [3], [4]. A standard CT imaging protocol usually includes nonenhanced and contrast-enhanced acquisitions [5]–[7]. Precontrast images are used as baseline density measurement of colon cancers, but also providing information about the presence of fat [8], necrosis, calcium [9], or hemorrhage [10]. Iodine-enhanced images can reveal the degree and pattern of tumor enhancement. Previous reports found that contrast-enhanced multidetector CT (MDCT) with multiplanar reformation (MPR) images could be used to assess suspected extensive colorectal cancers and identify invasion of pericolic fat planes and adjacent organs [5]–[7].

With recent advances in CT technology, dual-energy computed tomography (DECT) simultaneously acquires datasets at two different photon spectra in a single acquisition, and iodine can be distinguished from other materials owing to its stronger photoelectric absorption at low tube voltages near K-edge of iodine [11]. Current second generation DECT with tin filter technology improves not only the dual-energy ratio difference between iodine and calcium but also the spatial resolution and CT number homogeneity of VNE images in both phantom and in vivo experiments [12]. Image post processing algorithms based on three-material decomposition principles can create VNE images by removing the iodine-containing voxels [11], [13]. Furthermore, the dual-energy data can be used to generate a color-coded iodine overlay (IO) image that shows the distribution of iodine within the volume of tissue examined by CT [11]. This IO image can be superimposed onto VNE images for visualization of iodine distribution as well as anatomic information. If VNE images can replace TNE images, radiation dose and scanning time can potentially be reduced. Because patients with CRC require accurate preoperative CT staging for the planning of optimal therapy and repeated CT exams for follow up, efforts should be made to minimize the radiation dose of CT while preserving the diagnostic accuracy. The aim of this study was to evaluate whether the iodine overlay technique and VNE imaging by second generation DECT are effective for preoperative T-staging of CRC.

## Materials and Methods

### Patients

The Kaohsiung Medical University Hospital institutional review board approved the protocol of this retrospective study and issued a waiver of informed consent. Using a computerized search of our hospital's radiology records from January 2012 to July 2013 and the search term “staging of CRC,” we identified 162 patients with histopathologically proven CRC who had undergone preoperative CT for cancer staging in our institution. Based on the following criteria (i.e., the T-staging proven by surgery; patients who had undergone DECT, including TNE images and intravenous contrast enhanced images), we included 103 patients (67 men and 36 women; age range, 36–90 years [mean age, 65.8]). All patients underwent surgical resection within 14 days of CT examination. Pathologic findings for the depth of tumor invasion served as the reference standard. T-staging was based on the 2010 international TNM classification [14]. The mean body mass index of these patients was 24 kg/m^2^±3.5 (range: 16.4–35.6).

### CT Techniques

All CT scans were acquired using a 128-row dual-source, dual-energy MDCT scanner (SOMATOM Definition Flash; Siemens Medical Solutions, Forchheim, Germany) and tube current modulation software (CAREDose4D; Siemens Healthcare Sector) to adjust tube current in real time to maintain image noise at the optimal level [15].

Each patient drank 500 mL of water shortly before scanning. They were placed in the left lateral position, and a 12-F balloon-tipped rectal tube was inserted. A total of about 1200 ml of room air was gently insufflated into the colon. A standard CT scout image was obtained to assess the degree of colonic distention and deemed acceptable when all colonic segments were visualized and well distended. When necessary, further insufflation was performed. At first, a non-enhanced scan of the abdomen was acquired from the dome of the liver to the anal verge during a full-inspiratory breath-hold by using a detector configuration of 128×0.6 mm; rotation time, 0.5 second; pitch, 0.6; a tube potential of 120 kVp, and quality reference of 250 mAs. All patients received 100 mL of nonionic iodinated contrast agent (Ultravist 300; Bayer, Berlin, Germany), administered via the antecubital vein at 3 mL/sec by an automatic injector. Spiral DECT was performed in the portal venous phase (70 seconds) and the scan was acquired from the dome of the liver to the anal verge by tube A at 80 kVp, effective value of 300 mAs, and by tube B at Sn 140 kVp (140 kVp with tin filtering), effective value of 116 mAs. The scanning parameters of the DECT were: collimation, 128×0.6 mm; rotation time, 0.5 second; pitch, 0.6; tube A, 201–300 mAs; and Tube B, 82–110 mAs. The fields of view covered by tubes A and B were 50 and 33 cm, respectively.

### CT Post-processing, Image Reconstruction and Radiation Dose Assessment

The nonenhanced and enhanced portal venous phase DECT scans (80 and Sn 140 kVp images) were reconstructed by using a SAFIRE iterative reconstruction algorithm. Contiguous axial and coronal 5-mm TNE images and enhanced WA portal venous phase images were used for clinical interpretation.

The enhanced WA image approximates the 120 kVp image, which is automatically generated by combining 40% of the 80 kVp data with 60% of the Sn 140 kVp data as recommended by the vendor. All data sets were loaded onto a dedicated dual-energy post-processing workstation (syngo MMWP, version VE40A; Siemens Medical Solutions) and a data storage computer (syngo Plaza) with 1.5-mm section thicknesses and 1.0-mm reconstruction intervals for multiplanar reformations (MPRs). This process was fully automated and initiated by the CT technologist.

The commercially available software “Liver VNC” (syngo MMWP) was used to calculate iodine subtracted from contrast-enhanced images using a three-material mass fraction decomposition algorithm [11]. By assuming that every voxel in the abdomen is composed of fat, soft tissue, and iodine, the algorithm generates a map that encodes the iodine distribution in each individual CT voxel.

This map can subsequently be used to remove iodine from the image, resulting in a VNE image; or to color-code the iodine distribution, resulting in an iodine-only image. This iodine-only image was superimposed onto the VNE image, resulting in a color-coded iodine overlay (IO) image. The presence of iodine was indicated by the reddish color and the intensity of the color correlated with the relative amount of iodine content detected on the IO image. The IO can be superimposed with different percentages that range from 0 (VNC) to 100% (iodine only) along with the cancelation of non-iodine-containing voxels [11], [13], [16], [17]. The mean time required to generate both the IO and VNC images for a single patient was less than 1 min, which is convenient for practical clinical application.

For each patient, dose-length products (DLP) were recorded from the patient protocol. The estimated effective radiation dose values in millisieverts were obtained by multiplying the DLP by a conversion factor of 0.015 mSv⋅mGy⋅cm^−1^ [18].

### Quantitative Image Analysis

The quantitative image analysis was performed on 5-mm-thick axial CT images on a commercially available workstation (syngo MMWP, version VE40A; Siemens Medical Solutions). The TNE and VNE images were displayed side by side. The preset soft-tissue window (window width, 350 HU; window level, 40 HU) could be changed at will. For all measurements, the size, shape, and position of the region of interest (ROI) were kept constant among the two image sets by applying a copy and paste function at the workstation. All measurements were performed by a radiologist (M.C.S.) who knew the tumor site locations in all patients.

Mean CT numbers ± standard deviations of the normal reference tissues were obtained by using a circular ROI cursor. We calculated the attenuation of the aorta for a single ROI drawn in the vessel lumen; the attenuation of the main portal vein on three consecutive images; the mean attenuation of the liver from three ROIs (left lobe and anterior and posterior segments of the right lobe) on images obtained at the level of the main portal vein; the mean splenic attenuation using two ROIs, which were placed at the anterior and posterior aspect of the spleen; the mean attenuation of both kidneys using four ROIs, which were placed at the bilateral renal cortex at the level of the renal hilum; the mean attenuation of the pancreas using three ROIs, which were placed at the level of the pancreatic head, body, and tail; and the mean attenuation of the two paraspinal muscles using two ROIs, which were placed at each side without including macroscopic areas of fat. At all anatomical sites, the size of the ROI was maintained at approximately 1.0 cm^2^ (range, 0.3–1.5) if possible.

For the CRCs, the mean CT numbers ± standard deviations of the cancers on TNE, enhanced WA, and color-coded iodine overlay images were assessed by manually placing circular or freehand ROIs drawn to encompass as much of the tumor as possible (mean 1.8 cm^2^; range, 0.4–3.5). On color-coded iodine overlay images, the overlay value represents the average enhancement in Hounsfield units. Enhancement was defined as the difference of attenuation in the same region between enhanced WA image and TNE image. To ensure consistency, all tumor measurements were performed three times on each ROI, and mean values were calculated for each lesion.

Corresponding standard deviations were determined. Signal-to-noise ratio (SNR) was then calculated by dividing the mean CT number by the corresponding standard deviation [19]. For all CRCs, the maximal depth of each cancer was also measured with transverse images in both TNE and VNE protocols.

Quantitative image noise was measured as the standard deviation of the pixel values from a circular ROI(mean, 1.0 cm^2^, range, 0.8–1.2 cm2) drawn from the homogeneous subcutaneous fat of the anterior abdominal wall [20].

### Assessment of Local Cancer Staging

**Figure 1:**
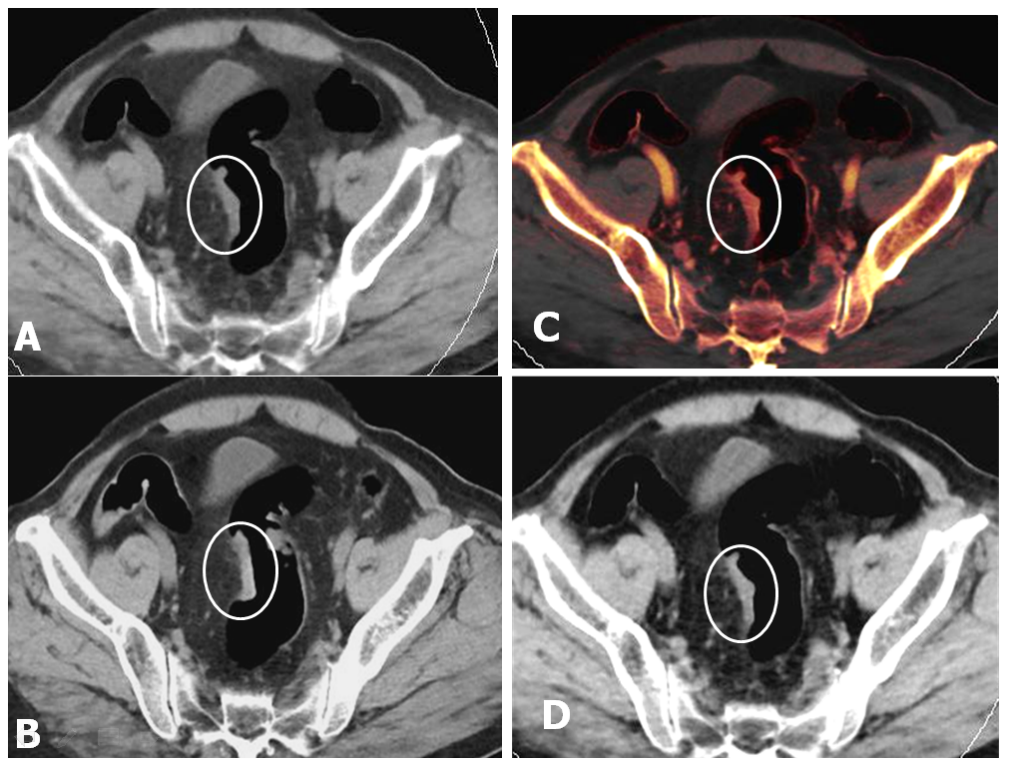
Axial images of the colon in an 80-year-old man (BMI = 27.7) with T1-2 sigmoid colon cancer. (A, B) VNE and TNE images show similar pictures of focal bowel wall thickening (white circle) in the sigmoid colon with smooth outer border. (C, D) 50% iodine overlay image and enhanced WA image during the portal venous phase show transmural enhancement (white circle) of the sigmoid colon cancer and smooth outer border. These findings suggest pathologic stage T1-2.

**Figure 2:**
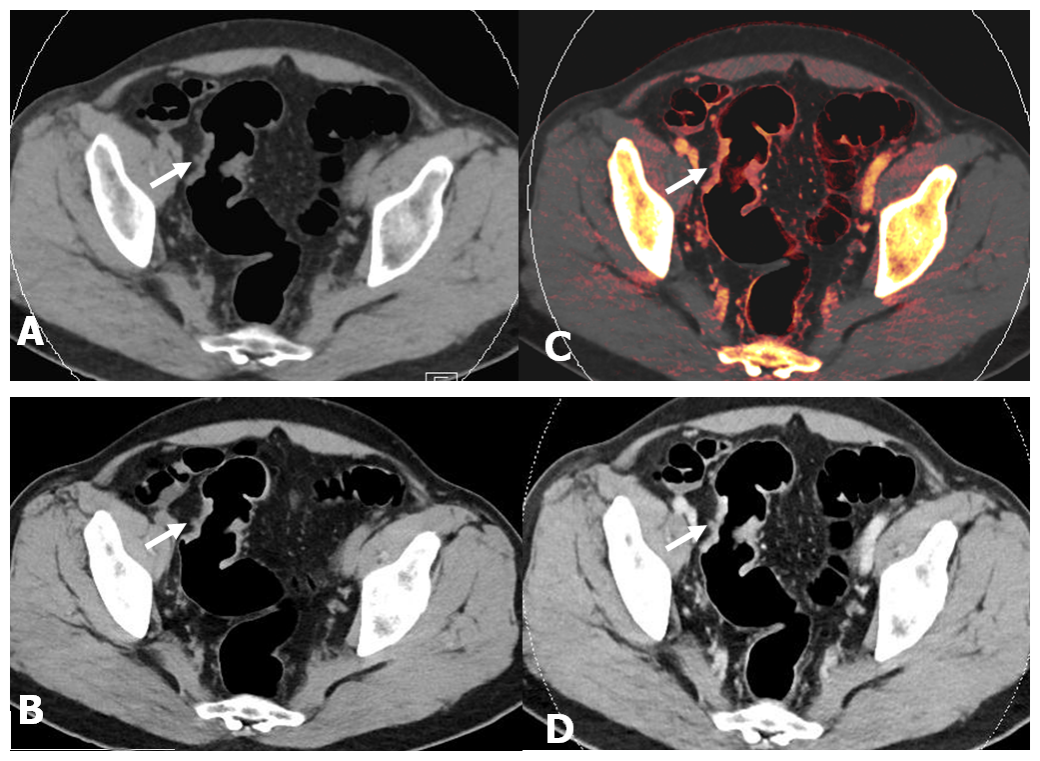
Axial images of the colon in a 51-year-old man (BMI = 25.1) with T3 sigmoid colon cancer. (A, B) VNE and TNE images show similar pictures of focal bowel wall thickening in the sigmoid colon with a nodular outer border (white arrow). (C, D) 50% iodine overlay image and enhanced WA image during the portal venous phase show transmural enhancement of the sigmoid colon cancer, an enhanced rounded advancing margin (white arrow) extending to the pericolonic fat. These findings suggest pathologic stage T3.

**Figure 3:**
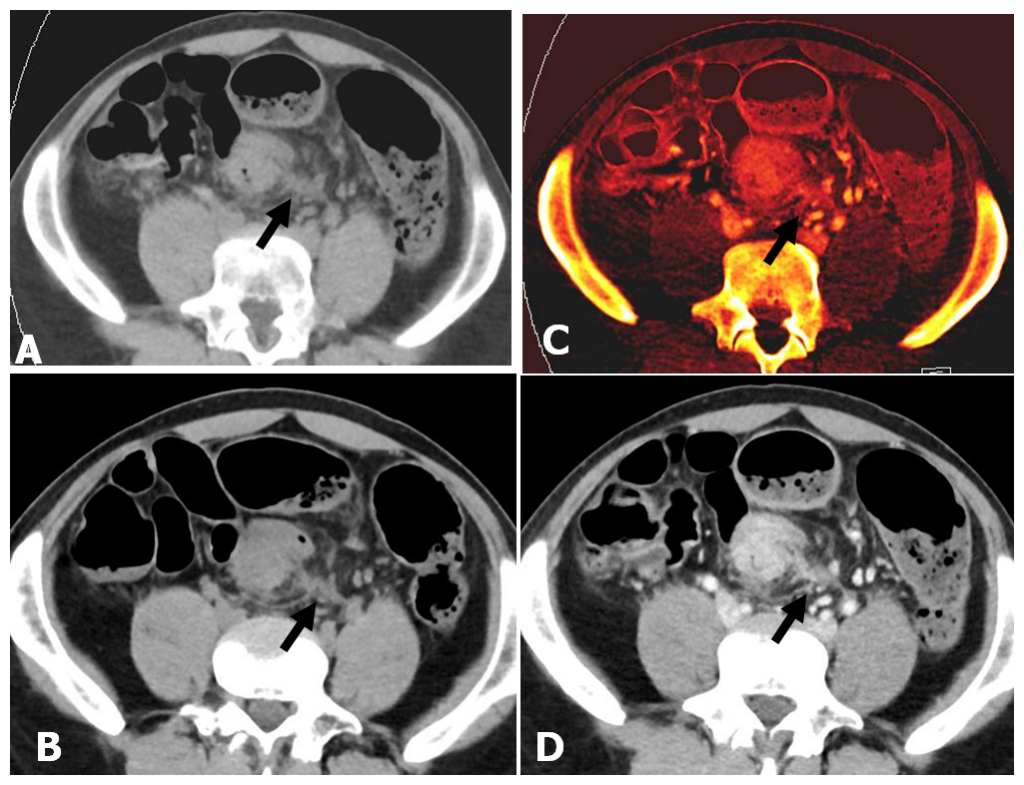
Axial oblique MPR images of the colon in a 47-year-old man (BMI = 24.7) with T4a sigmoid colon cancer. (A, B) VNE and TNE images show similar pictures of focal bowel wall thickening in the sigmoid colon with irregular outer border and obvious pericolonic fat infiltration. (C, D) Iodine image and enhanced WA image during the portal venous phase show transmural enhancement of the sigmoid colon cancer with irregular outer border, enhanced pericolonic fat infiltration, and direct invasion to the adjacent visceral peritoneum (arrow). These findings suggest pathologic stage T4a.

**Figure 4:**
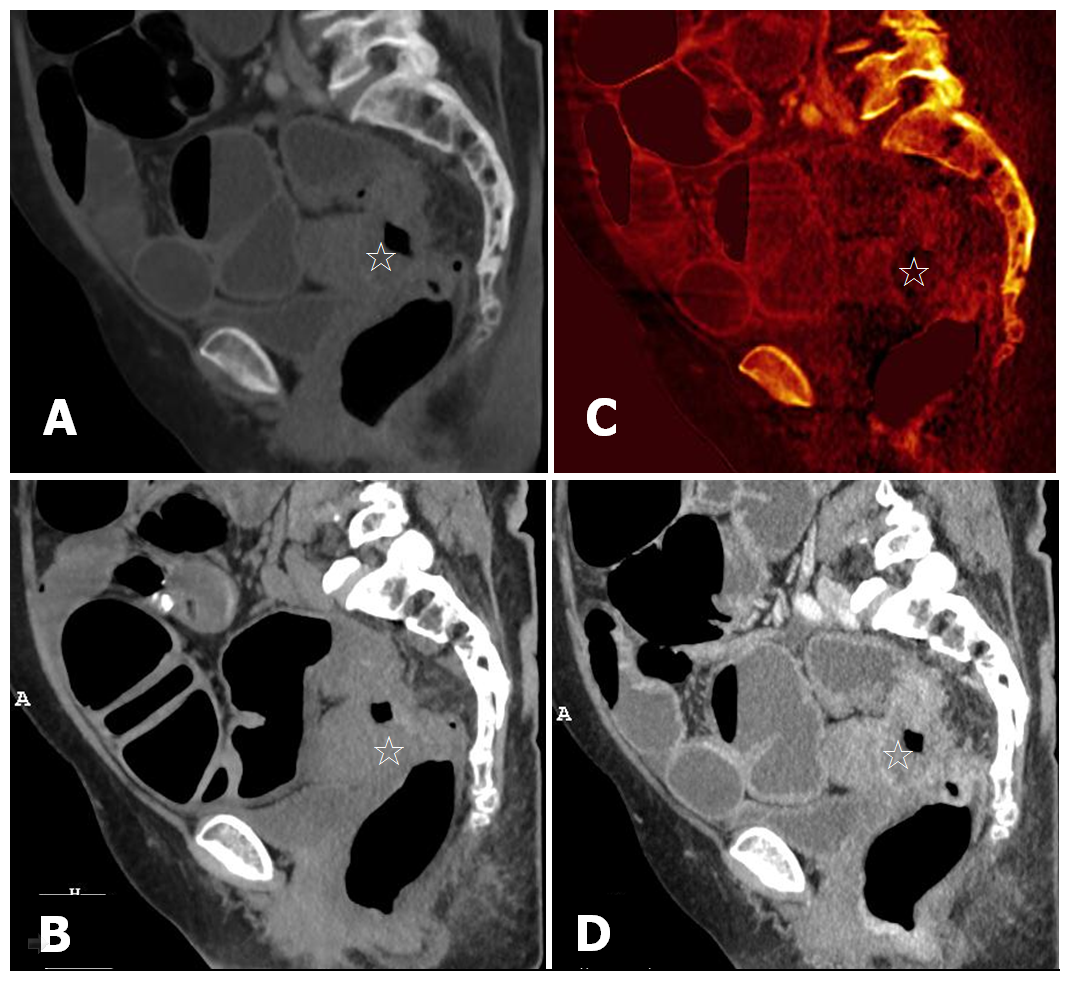
Sagittal oblique MPR images of the rectosigmoid colon in a 65-year-old woman (BMI = 31.1) with T4b rectosigmoid colon cancer. (A, B) VNE and TNE images show similar pictures of annular bowel wall thickening (star) in the rectosigmoid colon. (C, D) Iodine image and enhanced WA image during the portal venous phase show marginal enhancement of rectosigmoid colon cancer (star) with direct invasion into the posterior wall of the uterus. These findings suggest pathologic stage T4b.

Focal bowel wall thicker than the adjacent distended colonic wall; and the visualization of red color coded iodine within the lesion on IO image or significantly increased density of the lesion on the enhanced WA image were considered to indicate the presence of a neoplasm. Using MPR images, the depth of the tumor invasion was determined using a projection vertical to the tumor to avoid the partial volume effect. If a suspicious colon cancer was detected, according to a previous study, T1 and T2 tumors were combined to represent one T stage, ≤−T2. This classification was used to address CT's limitations in distinguishing T1 and T2 lesions [2]. According to the 2010 TNM system, the CT criteria for mural invasion of cancer into the colorectal wall are: (a) ≤−T2 stage: neoplasm shows focal thickening of colorectal wall with smooth outer wall border and clear fat plane around the tumor (Fig. 1); (b) T3 stage: transmural tumor with irregular or nodular outer border and/or pericolonic fat infiltration (Fig. 2); (c) T4a stage: transmural tumor with irregular outer border and pericolonic fat infiltration and direct invasion to the adjacent visceral peritoneum (Fig. 3); (d) T4b stage: obliteration of fat plane between CRC and adjacent organ or invasion of adjacent organ (Fig. 4).

Two experienced abdominal radiologists (T.S.J. and C.Y.C.), who were blinded to the location and stage of the CRCs, independently interpreted all dual energy CT scans in two separate reading sessions. In the first session, only images derived from the enhanced DECT acquisition (i.e., IO and VNE images) were assessed for the presence and sites of the CRC, as well as depth of CRC invasion (T-staging) and recorded the results for agreement analysis. After that, the two readers met and decided the final results for each case by consensus.

After 35–50 (mean, 42) days, simulated single-energy CT (SECT) was used to re-interpret cases. In this second reading session, TNE and enhanced WA images were used, thereby simulating a standard examination on a scanner without dual-energy capabilities.

### Qualitative Image Analysis

The other two experienced radiologists (G.C.L. and J.S.H.) independently ranked the quality of the TNE and VNE images using 5-mm-thick transverse CT images.

Image noise was rated on a visual analogue scale, which was subsequently converted to noise on a five-point scale: 1, *none*; 2, *minimal*; 3, *mild*; 4, *moderate*; and 5, *severe*. Overall image quality was rated on a different five-point scale: 1, *excellent*; 2, *good*; 3, *fair*; 4, *poor*; and 5, *noninterpretable*. The readers rated the presence of artifacts by using a 4-point scale, with 1 indicating *none*; 2, *mild*; 3, *moderate*; and 4, *severe*. The two radiologists finally decided (based on overall impression) whether VNE images were acceptable replacements for TNE acquisitions. Level of acceptance was rated 1, *completely*; 2, *partially*; and 3, *not* acceptable. In cases of inter-observer disagreement, final decisions were reached by consensus.

### Statistical Analysis

All numeric values are expressed as the mean ± standard deviation. Agreement in the CT numbers and SNRs between VNE and TNE images was analyzed using intraclass correlation coefficient (ICC) statistics. The paired *t* test was used to compare the image noise, the maximal tumor thickness, the iodine overlay and enhancement values of the tumor, and effective radiation dose. Test performance characteristics (sensitivity, specificity, and accuracy) of T-staging were calculated based on the 2 different paradigms for interpreting CT scans. Inter-observer variability between the two radiologist evaluators was evaluated using kappa (k) statistics. Definitions of agreement based on k values were as follows: <0.20, *slight agreement*; 0.20–0.40, *fair agreement*; 0.41–0.60, *moderate agreement*; 0.61–0.80, *substantial agreement*; 0.81–1.0, *almost perfect agreement*. All statistical analyses were performed with Stata, version 12. A *p*-value of less than 0.05 was considered to indicate a significant difference.

## Results

### Radiation Dose

Mean effective radiation exposure was 8.12 mSv±1.20 for the nonenhanced single-energy acquisition; 6.20 mSv±0.72 for the contrast-enhanced dual-energy acquisition; and 14.32 mSv±1.91 for the biphasic protocol. Omitting of the TNE phase, the radiation dose could be decreased by 57% with the single-phase dual-energy CT protocol (i.e., IO and VNE images) only.

### Quantitative Image Analysis

There was significant correlation (though not concordance) between TNE and VNE images in CT numbers of the tumor, aorta, psoas muscle, and pancreas (ICC: 0.29, 0.50, 0.36, 0.69, respectively; *P*<.01, Table 1) and in SNR of the tumors and normal reference tissues (*P*<.01). The mean iodine overlay value (48.4 HU±12.2) and enhancement (49.4 HU±11.8) value of CRCs were not significantly different (P = 0.52). No significant difference existed in the mean image noise between TNE (5.0±1.1) and VNE (5.3±1.1) images (*P* = 0.07).The transverse-section maximal tumor thickness on TNE (1.6 cm±1.1) and VNE (1.6 cm±1.1) images (*P* = 0.99) also showed no significant difference.

**Table 1:** Quantitative Image Analysis of Normal Reference Tissues and Tumors in TNE and VNE CT Images.

	TNE	VNE	ICC^a^	*P* value^a^
	Mean±SD	Mean±SD		
CT number (HU)				
Liver	52.8±4.8	63.9±6.2	−0.117	0.882
Spleen	44.2±3.4	61.9±4.7	−0.775	1.000
Aorta	40.5±4.4	43.4±5.2	0.502*	<0.001
Fat	−107.4±8.7	−91.9±7.9	−0.094	0.828
Muscle	49.3±6.1	57.0±7.0	0.358*	<0.001
Pancreas	41.7±6.4	45.7±7.0	0.686*	<0.001
Kidney	29.7±2.7	37.4±3.8	−0.359	1.000
Tumor	33.9±8.1	38.0±8.8	0.286	0.002
Signal to noise ratio				
Liver	8.9±2.1	9.6±2.0	0.752*	<0.001
Spleen	7.9±2.1	10.0±1.8	0.294*	0.001
Aorta	6.0±1.2	5.7±1.1	0.643*	<0.001
Fat	−23.1±5.5	−18.6±4.4	0.459*	<0.001
Muscle	7.9±2.3	8.8±2.3	0.794*	<0.001
Pancreas	6.7±1.8	6.7±1.9	0.798*	<0.001
Kidney	5.2±1.3	5.8±1.3	0.705*	<0.001
Tumor	5.1±2.1	5.4±2.1	0.764*	<0.001
Image noise (HU)	5.0±1.1	5.3±1.1	0.813*	<0.001

Abbreviations: TNE, true nonenhanced, VNE, virtual nonenhanced; CT, computed tomography; ICC, intraclass correlation coefficient; HU, Hounsfield units.

Note. –Data are mean values ± standard deviation.

a ICC was calculated for the intraclass correlations between VNE and TNE.

*P* value denotes the statistical significance for ICC.

### Local tumor staging

**Figure 5:**
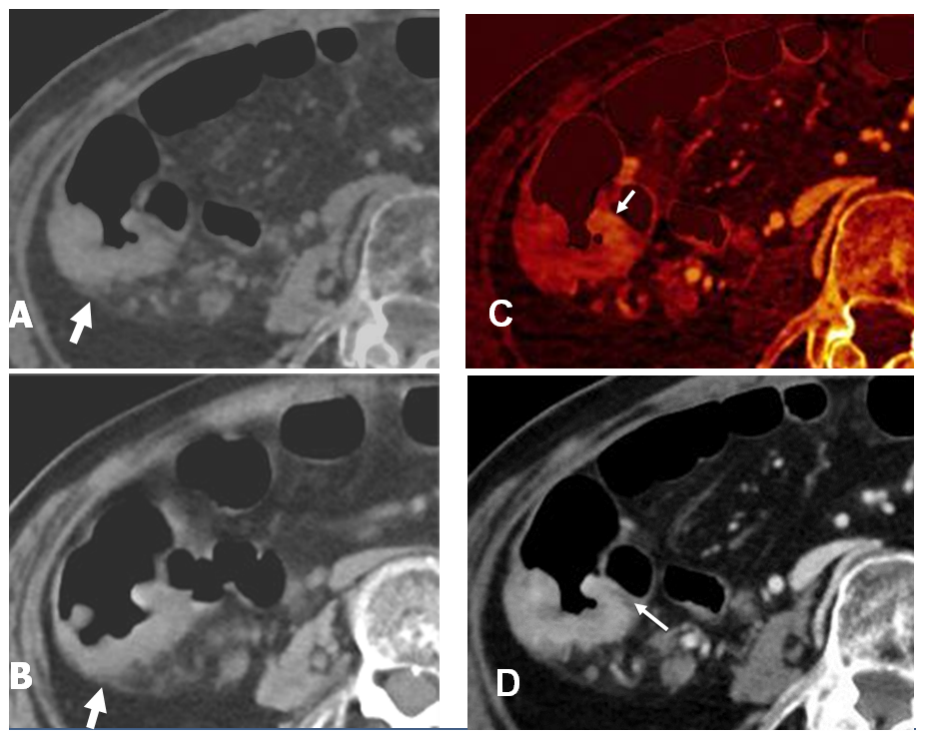
T4b cancer correctly staged by single-phase protocol and mistakenly classified as T4a by dual-phase protocol in an 87-year-old female (BMI = 22.7). (A, B) VNE and TNE axial images show similar pictures of focal bowel wall thickening in the cecum with irregular outer margin and pericolonic fat infiltration into the visceral peritoneum (white arrow). (C, D) Axial view of iodine image and enhanced WA images during the portal venous phase show transmural enhancement of the tumor with irregular outer margin. Focal red-colored enhanced tumor direct invasion into the adjacent wall of the terminal ileum (white arrow) is well demonstrated on iodine image, but not visualize on the enhanced WA image. The preserved fat plane between the cancer and terminal ileum is erroneously interpreted on the enhanced WA image (white arrow). These findings suggest stage T4b by the single phase protocol and stage T4a by the dual phase protocol. An invasion of the terminal ileum was proved by the pathologic staging (pT4b).

**Figure 6:**
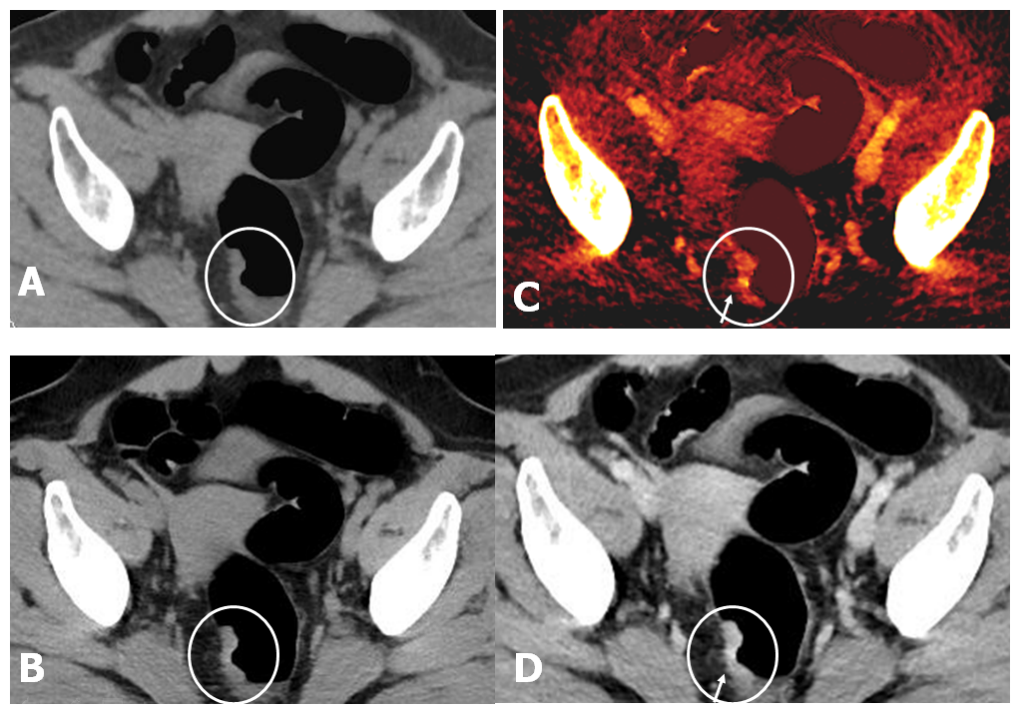
T3 cancer correctly staged by single phase protocol and mistakenly classified as T1-2 by dual phase protocol in a 48-year-old female (BMI = 25.2). (A, B) VNE and TNE axial images show similar pictures of focal bowel wall thickening (white circle) in right side of the rectosigmoid colon with smooth outer border. (C, D) Iodine image and enhanced WA image during the portal venous phase show transmural enhancement of the tumor (white circle). Focal red-colored enhanced tumor direct invasion into the adjacent pericolic fat is well demonstrated on iodine image (white arrow), but not visualize on the enhanced WA image (white arrow). These findings suggest stage T3 by the single phase protocol and stage T1-2 by the dual phase protocol. The pericolonic fat infiltration was proved by the pathologic staging (pT3).

The detection rates of CRCs by using MPR images were 100% (103 of 103 neoplasms) with 20 neoplasms (19%) at the ascending colon, 6 (6%) at the transverse, 13 (13%) at the descending, 60 (58%) at the sigmoid, 3 (3%) at the rectosigmoid colon, and 1 (1%) at the rectum. At the histopathologic examination, 28 of 103 neoplasms (27%) were staged as pT1-2 (Fig. 1), 66 (64%) as pT3 (Fig. 2), 7 (7%) as pT4a (Fig. 3), and 2 (2%) as pT4b (Fig. 4). The overall diagnostic accuracies of T staging with MPR images was better in single-phase DECT (VNE and IO images) than in dual-phase simulated SECT (TNE and enhanced WA images) (90.3% [93 of 103 neoplasms] vs. 87.4% [90 of 103 neoplasms]) (*p* = 0.51) (Fig. 5, 6). Over- and under-staging occurred in 8 (8%) and 5 (5%) patients, respectively, using the dual- phase protocol; and in 8 (8%) and 2 (2%) patients, respectively, using the single-phase protocol. Accuracy rates of T-staging with dual-phase and single-phase images were 93% and 95% for T1-2, 89% and 90% for T3, 94% and 95% for T4a, and 98% and 100% for T4b, respectively (Table 2). Kappa values for the agreement between imaging and pathological diagnosis obtained from readers 1 and 2 were 0.85 and 0.77 for single-phase and dual-phase, respectively (*p*<0.05).

**Table 2:** Diagnostic Accuracy of Dual-phase and Single-phase CT Images for Each T Stage with Histopathologic Results as Reference Standard.

	Histopathologic Stage^a^				Accuracy, %	Sensitivity, %	Specificity, %
	T1-2	T3	T4a	T4b			
Dual-phase							
T1-2	24	3	0	0	93.2	85.7	96.0
T3	3	59	1	0	89.3	89.4	89.2
T4a	0	4	6	1	94.2	85.7	94.8
T4b	1	0	0	1	98.1	50.0	99.0
Kappa^b^: 0.772*							
Single-phase							
T1-2	24	1	0	0	95.2	85.7	98.7
T3	4	61	1	0	90.3	92.4	86.5
T4a	0	4	6	0	95.2	85.7	95.8
T4b	0	0	0	2	100.0	100.0	100.0
Kappa^b^: 0.852*							

Abbreviations: Dual-phase: Enhanced WA 120-kVp and TNE images; Single-phase: Iodine Overlay and VNE images.

Note.—Overall accuracy of T staging was 87.4% (90 of 103 neoplasms) with dual phase and 90.3% (93 of 103 neoplasms) with single phase CT images (*P* for difference: 0.507).

a Data are numbers of neoplasms.

b Kappa value denotes the agreement of pathological diagnosis between reader 1 and reader 2, and * denotes significant agreement (*P*<0.05).

### Qualitative image analysis

In the visual evaluation, the overall image quality of VNE and TNE images was excellent in 102(99%) and 46 (45%) patients, and good in 1(1%) and 57(55%) patients, respectively. The overall image quality scores were relatively better for TNE images (1.0±0.1) than for VNE images (1.6±0.5) (Table 3, *P*<0.05). In both groups, image noise was ranked *no* or *minimal*, and artifact was ranked *none* or *mild*.The mean image noise score was 1.70±0.05 for VNE and 1.1±0.04 for TNE images (*P*<0.05). More artifacts were found on VNE images (1.5±0.6) than on TNE images (1.1±0.4), including rim artifacts at the dome of the liver [n = 27(19%) and 2(2%)], streak artifacts [n = 20(19%) and 7(7%)], and severe beam-hardening artifact from a metal prosthesis of the spine (n = 1 and 1), respectively. VNE images were *completely* acceptable for replacement of TNE images in all patients by both reviewers. The agreement between the two reviewers was *substantial* or *almost perfect agreement* (Kappa value>0.6, Table 3).

**Table 3:** Image Quality of the TNE and VNE Images.

Qualitative scoring^a^	TNE	VNE	Difference
**Scoring** a **, mean±SD**			
Overall image quality (1–5)	1.0±0.1	1.6±0.5	–0.6*
Image noise (1–5)	1.0±0.01	1.7±0.05	–0.7*
Image artifacts (1–4)	1.1±0.04	1.5±0.6	–0.4*
Level of acceptance (1–3)	1.0±0.0	1.0±0.0	0.0
**Kappa value** b			
Overall image quality (1–5)	1.000*	0.802*	
Image noise (1–5)	NA	0.781*	
Image artifacts (1–4)	0.828*	0.604*	
Level of acceptance (1–3)	1.000*	1.000*	

Abbreviations: TNE, true nonenhanced, VNE, virtual nonenhanced; NA, non-appreciable.

a Image quality is scaled as 1 to 5 (1 *excellent*, 2 *good*, 3 *fair*, 4 *poor*, 5 *noninterpretable*); image noise is scaled as 1 to 5 (1 *none*, 2 *minimal*, 3 *mild*, 4 *moderate*, 5 *severe*); image artifacts is scaled as 1 to 4 (1 indicating *none*, 2 *mild*, 3 *moderate*, 4 *severe*); level of acceptance is rated as 1 to 3 (1 *completely*, 2 *partially*, 3 *not* acceptable).

b Kappa value denotes the agreement of image scoring between reader 1 and reader 2.

* denotes *P*<0.05 for differences in qualitative scores between TNE and VNE, and for agreements of image scoring between reader 1 and 2.

## Discussion

Our results show that single-phase DECT using color-coded IO and VNE images provide high accuracy in T-staging of CRC and suggest that VNE images could potentially replace TNE images. This change would reduce patient exposure to radiation of CT. The superior overall accuracy of T staging with VNE and IO images to that with TNE and enhanced WA images (90.3% vs 87.4%) might help clinicians optimize the treatment regimens for individual cases.

The IO images using the color-coded mapping may improve visual delineation of lesions. Because color representing the concentration of enhanced iodine is encoded on original CT images, excellent anatomic detail is preserved and lesions can be easily discriminated from their surroundings. Boellaard et al concluded that detection of CRC is feasible at DECT without bowel preparation or air insufflation after they found a diagnostic accuracy of 90% (27/30) of the cancers with 120 kVp images only and 96.7% (29/30) with viewing the iodine map in addition [21]. Hence, CT is currently the standard modality for staging CRCs before curative surgical resection [1], [5]–[7]. This study demonstrated 100% detection rate of CRC in our patients with room air insufflation. All patients tolerated well for the procedure.

With advances in CT technology and computing software, CT has shown potential not only as a staging tool but also in predicting the prognosis of CRC [1], [2]. There has also been of interest recently in developing neoadjuvant treatment strategies for patients with colon cancer because of better compliance and potential to downstage prior to surgical treatment [3], [4]. The key feature in predicting prognosis in CRC is the extent of tumor spreading beyond the muscularis propria [1], [2], [5]. Therefore, there is a need to select the best CT techniques for staging. DECT provides direct visualization of iodine uptake within tumor in color-coded fashion, which makes a reliable quantification of enhancement without HU measurements. Morrin et al. [7] found that contrast-enhanced MDCT was helpful in assessment of the patients suspected of having extensive CRC; it permitted identification of invasion of pericolic fat planes and the adjacent organs. The IO images generated by DECT can well demonstrate the extracolonic spread of the tumor, because IO images can display iodine concentration and distribution in the tumor and surrounding tissues with red-color encoding. In this study, the mean iodine overlay value and enhancement value of CRCs showed no significant difference. Furthermore, we found that combined analysis of IO and VNE images may also be useful for evaluating extracolonic spread of advanced CRCs. Our study showed that the sensitivity in T-staging of T3 (Fig. 5) and T4b (Fig. 6) lesions on IO images was better than on WA-enhanced images.

Several previous studies have reported that VNE images are reasonable approximations to their TNE counterparts in patients with renal masses [8], [22], [23], liver lesions [24], adrenal masses [25], gallstones or bile duct stones [26] as well as urinary stones [27], [28]. Obtaining VNE images may decrease the need for TNE images, thereby reducing radiation dose. In our study, the maximal thickness and SNR of the CRC were similar on VNE and TNE images. Qualitative image noise and image quality scores were rated slightly poorer on VNE images than those of TNE images. However, in this study the difference in image quality did not affect the diagnostic value of VNE images, and the two experienced abdominal radiologists found both images fully acceptable.

In this study, tin filtration was applied on the high-energy x-ray tube of DECT scanner. A recent study reported that adding tin filtration to the high kVp tube not only increases the dual-energy contrast between iodine and calcium, with radiation dose being similar to or lower than that of conventional single-energy CT, but also decreases noise in the VNE images in both phantom and in vivo experiments [12]. We found that the iodine components could be successfully removed from the enhanced soft tissues to create VNE images. There were reliable agreements between TNE and VNE images in CT numbers of the tumor and normal reference tissues. If the TNE images are replaced by VNE images, the additional radiation exposure could be avoided.

From this study, color-coded IO and VNE images derived from contrast-enhanced DECT data can be used to adequately detect and stage CRC. Decreasing radiation exposure as low as reasonably achievable (ALARA) is one of our clinical objectives and it is also an important advantage of DECT for patients with CRC. To our knowledge, no studies concerned with detection and staging of colorectal cancers by DECT have been published in the literature. At our institution, just like some other institutes, we used to have a nonenhanced and a contrast-enhanced acquisition for assessment of CRC. If the non-contrast scan would be omitted, the dose could reduce from 14.3 mSv to 6.2 mSv.

Several potential limitations of our study merit consideration. First, due to the retrospective nature of our study, nonenhanced scan was performed with a standard dose protocol (120 kVp; quality reference, 250 mAs) in our department. As dose is proportional to tube current, a reduction in tube current can also cause a significant decrease in patient dose. In this study, we used a conventional TNE imaging rather than low-dose imaging as a high standard to evaluate whether the imaging quality of the VNE could be good enough to obviate the need of TNE acquisitions and thus reducing the radiation dose of the patients. Second, our conclusions are based on WA 120-kVp images obtained on a dual-energy CT scanner, rather than conventional 120-kVp acquisition. We did not perform an additional 120-kVp scan because it would have exposed the patients to additional radiation. Nevertheless, the weighted CT data obtained at various photon energies is expected to be used with increasing frequency in the future, whenever DECT is available. Third, though reading order can impact evaluation of VNE images, readers cannot be completely blinded to the image sets, as the difference between sets is obvious. Finally, we did not evaluate the N and M staging. Further studies evaluating the N and M staging are therefore warranted.

In conclusion, the single-phase protocol using iodine overlay and VNE images by second generation DECT yields good preoperative T-staging of CRC. The VNE images may decrease the need for TNE images, thereby, the radiation exposure of the patients can be reduced.
